# District Health Officer Perceptions of PEPFAR’s Influence on the Health System in Uganda, 2005-2011

**DOI:** 10.15171/ijhpm.2016.98

**Published:** 2016-07-26

**Authors:** Nathaniel Lohman, Amy Hagopian, Samuel Abimerech Luboga, Bert Stover, Travis Lim, Frederick Makumbi, Noah Kiwanuka, Flavia Lubega, Assay Ndizihiwe, Eddie Mukooyo, Scott Barnhart, James Pfeiffer

**Affiliations:** ^1^Department of Global Health, University of Washington, Seattle, WA, USA.; ^2^Department of Health Services, University of Washington, Seattle, WA, USA.; ^3^Faculty of Health Sciences, Makerere University, Kampala, Uganda.; ^4^Division of Global HIV and Tuberculosis, Atlanta, GA, USA.; ^5^Resource Center for the Uganda Ministry of Health, Uganda Ministry of Health, Nakasero, Uganda.

**Keywords:** President’s Emergency Plan for AIDS Relief (PEPFAR), Health System Strengthening, Global Health Initiatives (GHIs), District Health Officers (DHOs), Uganda, HIV

## Abstract

**Background:** Vertically oriented global health initiatives (GHIs) addressing the HIV/AIDS epidemic, including the President’s Emergency Plan for AIDS Relief (PEPFAR), have successfully contributed to reducing HIV/AIDS related morbidity and mortality. However, there is still debate about whether these disease-specific programs have improved or harmed health systems overall, especially with respect to non-HIV health needs.

**Methods:** As part of a larger evaluation of PEPFAR’s effects on the health system between 2005-2011, we collected qualitative and quantitative data through semi-structured interviews with District Health Officers (DHOs) from all 112 districts in Uganda. We asked DHOs to share their perceptions about the ways in which HIV programs (largely PEPFAR in the Ugandan context) had helped and harmed the health system. We then identified key themes among their responses using qualitative content analysis.

**Results:** Ugandan DHOs said PEPFAR had generally helped the health system by improving training, integrating HIV and non-HIV care, and directly providing resources. To a lesser extent, DHOs said PEPFAR caused the health system to focus too narrowly on HIV/AIDS, increased workload for already overburdened staff, and encouraged doctors to leave public sector jobs for higher-paid positions with HIV/AIDS programs.

**Conclusion:** Health system leaders in Uganda at the district level were appreciative of resources aimed at HIV they could often apply for broader purposes. As HIV infection becomes a chronic disease requiring strong health systems to manage sustained patient care over time, Uganda’s weak health systems will require broad infrastructure improvements inconsistent with narrow vertical health programming.

## Background

More than 10 years now since its inception, there is general consensus the US President’s Emergency Plan for AIDS Relief (PEPFAR) has been successful at its main goal – to reduce the morbidity and mortality from HIV/AIDS in targeted low-income countries.^1,2^ Among other positive effects specifically pertaining to HIV, PEPFAR increased equity and access to antiretroviral treatment (ART), raised standards of care, and improved diagnostic laboratory capacity.^3-6^ However, the jury is still out as to whether PEPFAR investment contributed to strengthen, perhaps undermined, or had no effect on broader health systems. Did PEPFAR provide much needed funding, energy, and support to health systems in countries where it operated, either by “spilling-over” resources from its predominantly vertical HIV/AIDS programs, or by directly investing in health system strengthening beginning in 2008, as some have hypothesized?^7,8^ Conversely, did PEPFAR undermine health systems, even if unintentionally, by recruiting health workers away from primary care duties, distracting health decision-makers, establishing parallel service delivery and reporting structures, and prioritizing HIV/AIDS over potentially more pressing challenges, as others have suggested?^5,6,9-12^

Studies attempting to investigate PEPFAR’s influence on health systems offer mixed evidence for productive and counterproductive effects. Some researchers have observed positive effects on primary care,^8,13^ increased vaccination rates, antenatal care coverage, malaria diagnoses, and treatment of sexually transmitted diseases.^13-16^ There is evidence, however, health service providers have shifted focus from primary care duties, leading some to challenge the reports of positive PEPFAR spillover.^11,12,17^ While some studies have demonstrated PEPFAR was associated with effective task-shifting, improved in-service training, and higher job satisfaction,^5,6,18^ other studies have noted an internal brain-drain of doctors from the public sector to HIV programs run by non-governmental organizations (NGOs), work interruption for training, and overburdening of a health workforce already spread thin.^3-6,9,17,19^

Despite this burgeoning body of literature analyzing the interactions between PEPFAR and health systems, few researchers have asked public sector health managers in low-income countries what they think about how PEPFAR influenced the health system. This is a noteworthy shortcoming because their views could valuably inform PEPFAR policy. Studies which have included analyses of stakeholder experiences have relied on a small number of interviews in a limited geographical area,^16,20^ focused on the experiences of patients^21^ and policy-makers,^6,9^ or assessed the marginalization of civil society organizations.^22^ Many studies have avoided distinguishing between PEPFAR, the Global Fund, and the World Bank’s Multi-Country HIV/AIDS Program, and have instead examined the net effects of global health initiatives (GHIs) generally.^5,19,23^ Those studies which have assessed a single initiative have tended to focus on the Global Fund.^24-39^ This is an important distinction because there are valid reasons to believe the varied policies and approaches of each GHI may have interacted with health systems in distinct ways.^40^

At the outset of PEPFAR in 2004, 960 000 people were living with HIV in Uganda,^41^ of whom fewer than 50 000 were receiving life-saving ART (Table 1). Uganda experienced significant population growth,^42^ and it had, and continues to have, an acute shortage of health workers^43^ and low domestic investment in health at 11% of public expenditure, or $52 per capita.^44^ PEPFAR implementing agencies initially made a strategic decision to rapidly expand HIV care and treatment services in order to save lives and control the spread of the epidemic. To do so as quickly as possible, PEPFAR created and used HIV-specific systems when existing government systems were deemed insufficient, such as parallel supply chain systems, funding mechanisms, information systems, and monitoring and evaluation (M&E) processes.^9,26,45^ The majority of programmatic funding flowed to implementing partners, mostly US-based NGOs, with little direct financial support allocated to Ugandan government health budgets. These programs slowed HIV incidence and significantly expanded access to ART. By 2010, more than 200 000 HIV-positive Ugandans were receiving treatment (Table 1), and by 2015 this figure had grown to an estimated 740 000.^46^ During subsequent funding phases, particularly 2008-2013, PEPFAR and other GHIs updated their strategies to include funding intended to strengthen the health systems required to sustain a long-term HIV response.^22,47,48^ However, in Uganda as in other PEPFAR “focus countries,” it remains an open question whether PEPFAR has strengthened, weakened, or had little effect on the overall health system.

To address this knowledge gap, our study gathered textual data characterizing District Health Officers’ (DHOs) perceptions of how PEPFAR affected health system strength in Uganda 2005-2011. We hypothesized Ugandan DHOs would perceive PEPFAR strengthened the health system overall, particularly in the areas of health workforce trainings, increased access to medical supplies and equipment, and improved financing for the health sector. We also hypothesized DHOs would perceive PEPFAR implementation prompted the movement of health workers out of the public sector and focused attention on HIV programming at the expense of primary healthcare delivery. Therefore, DHOs would have mixed views of PEPFAR’s influence on the health system.

To our knowledge, this is the first survey assessing the effects of PEPFAR on the experiences of DHOs across an entire country. Moreover, because contributions from PEPFAR were much larger than from other GHIs, constituting 73% of Uganda’s budget for HIV activities and more than a quarter of total health sector funding in 2006 as reported Oomman et al,^40^ and outpacing both Ugandan government health allocations and contributions from other GHIs each year 2005-2010 (Table 1), this study inherently focuses more on the effects of PEPFAR than many previous analyses.

**Table 1:** Ugandan ART Patient Volume and Health Sector Funding by Source 2005-2010

	**2005**	**2006**	**2007**	**2008**	**2009**	**2010**
ART patients^a^	49 638	51 397	83 549	130 837	175 367	207 872
Ugandan government allocation to health sector^b^ ($ inmillions)	129.2	132.2	164.6	226.4	217.9	258.9
Donor projects and other GHIs^b^ ($ in millions)	150.8	75.9	83.8	152.6	147.7	41.5
PEPFAR donor contributions^c^ ($ in millions)	146.9	170.0	236.6	283.6	285.9	286.3

Abbreviations: ART‏, antiretroviral treatment; GHIs, global health initiatives; PEPFAR, President’s Emergency Plan for AIDS Relief. Data sources: ^a^Uganda Monitoring and Evaluation of the Emergency Plan Progress (MEEPP) data provided by Social and Scientific Systems, Inc. ^b^Uganda’s Annual Health Sector Performance Report 2010/2011, page 26 Table 15. ^c^US Government Accountability Office, Report to Congressional Committees, PEPFAR, September 2010 GAO-10-836.

Ugandan DHOs’ perceptions of PEPFAR are necessary to determine whether the vertical strategy for HIV programs helped or hurt the health system as a whole. Though PEPFAR in Uganda is largely a vertical program outside the direct control of DHOs or the Ministry of Health (MoH), DHOs are responsible for managing the delivery of public sector health services for both HIV and non-HIV care. Moreover, given the Ugandan context of a shift towards decentralized health authority and decision-making, DHO roles are increasingly important.^5,22,40,48,49^

## Methods

This mixed-methods analysis of Ugandan DHOs’ perceptions about the positive and negative effects of PEPFAR is part of a larger evaluation of the influences PEPFAR had on the Ugandan health system between 2005-2011. For this paper, we collected quantitative and qualitative data through semi-structured interviews with leaders at each Ugandan district health office. After obtaining written consent to participate, we first asked each DHO to categorize whether PEPFAR’s influence on seven separate health system components (fully described below) had been positive, negative, or neutral. We then prompted DHOs to provide comments on each component. We had 30 to 60 minutes of the DHOs’ time to complete the questionnaire. During analysis, we used the categorical responses to complement the positive and negative comments each DHO offered and determine how much emphasis to place on each comment. We established the relative salience of positive and negative comments concerning each health system component based upon: (1) the number of DHOs making similar remarks and (2) the relative proportion of DHOs who categorized PEPFAR’s effects on that health system component as positive, negative, or neutral.

The project was funded with a PEPFAR Public Health Evaluation award to the University of Washington (UW) from the US Centers for Disease Control (CDC) in late 2010. Prior to issuing the award, the health systems team in the Health Economics, Systems and Integration Branch, Division of Global AIDS, at CDC had outlined a proposed research protocol. The UW, in partnership with Makerere University and CDC, prepared the final research protocol, including sampling strategy and analysis plan. Makerere University, Kampala, Uganda provided in-country implementation including leadership, project management, development of the questionnaire, and data collection. The Uganda office of the CDC and the Resource Center at the Ugandan MoH also made valuable contributions to the research.

Makerere University hired research assistants to collect data from the 112 district health offices. Recent university graduates, many with degrees in health and social sciences, comprised most of the team, along with some junior faculty. We conducted a one-week training in October 2011 to orient our team to the goals and the research protocol and provided a field manual outlining data collection procedures (manual available upon request).

Following the training, the research assistants were divided into six teams and assigned a geographical region. Each team consisted of three data collectors, one of whom was the designated leader. During the first week of data collection, each team visited and gathered data from nine district health offices and submitted these data to the research leadership team at Makerere University to be reviewed for thoroughness and to troubleshoot any problems. The teams then returned to the field and completed data collection from the district health offices by late December 2011.

District health offices were informed of the research project in advance by a letter from the MoH authorizing participation in the research. The research assistants then contacted each district health office to schedule their visit, confirmed their appointment with a call the day prior to arrival, and carried a letter of introduction from the MoH. In addition to the interviews with DHOs, we collected quantitative data from the routine MoH health management information system (HMIS) reports (fully described in Luboga et al^50^). Consent forms promised DHOs their identities would be kept confidential in our reports.

We conducted semi-structured qualitative interviews with DHOs to gather their perceptions of PEPFAR’s effects on health system components. If DHOs were unavailable, instructions prompted interviewers to speak with “the most senior district health officials or someone very knowledgeable about the district health office.” Table 2 details the professional titles and years of experience in their current district of the respondents. Our interview guide is available upon request. The team leaders administered the interview while a second member wrote responses on the interview guide. We determined handwritten responses were sufficient, and audio recording was unnecessary because our research question was neither interpretive nor reliant on the precise tone or syntax of the responses. All members of the data collection teams had been trained to take accurate notes and probe respondents for rich responses. Paper copies of the completed interview guides were scanned in the field, and the electronic copies were uploaded into a secured project “Dropbox.” Team members then transcribed the responses from the handwritten copies to digital format. These electronic transcripts were also uploaded to the password-protected “Dropbox,” where UW and Makerere researchers accessed them for analysis.

**Table 2:** Survey Respondents Categorized by Professional Title

**Professional Title of Respondent**	**No. of Respondents**	**Years in District** **Median (Range)**
DHO	74	4.0 (<1–21)
Assistant DHO	12	5.5 (<1–18)
District Health Educator	8	6.0 (1–20)
District Health Inspector	7	11.0 (1–25)
HIV/AIDS Focal Person	4	6.0 (1–22)
Senior Clinical Officer	3	4.0 (1–25)
Medical Superintendent	2	1.0
Public Health Nurse	1	10.0
Missing	1	1.0
Total	112	4.5 (<1–25)

Abbreviation: DHO, District Health Officer. Data source: Interviews with Ugandan DHOs (or their proxies) in 112 districts between October and December, 2011.

We used the World Health Organization’s (WHO’s) six building blocks for strong health systems^51^ to structure the questionnaire topics including the capacity to plan, monitor and evaluate, manage medical equipment, develop human resources (HRs), coordinate stakeholders in the health system, mobilize financial resources, and manage information systems. In this paper, we refer to these key areas of the health system as “components.” The interview guides included items that asked: (1) whether informants thought PEPFAR investment had improved, had no effect on, or decreased each component of the health system as related to the non-HIV health system; (2) to list and explain a few ways in which the PEPFAR investment had both positively and negatively affected each key component as it related to non-HIV health service delivery, regardless of whether their overall impression was of improvement, neutrality, or reduction; (3) to state whether their district had completed a number of key exercises pertaining to each health system building block in 2005/2006, 2006/2007, or 2010/2011; (4) to hypothesize two or three potential explanations why the quantitative aspects of this program analysis might find the PEPFAR investment had positive or negative effects on non-HIV health indicators; (5) to make any comments they would like to be included in the study.

Using the scanned copies of the structured interview guides containing the handwritten responses from each interview, we confirmed the electronic transcriptions entered into CSPro in the field were complete and accurate. In the instances where the electronic transcripts had been misentered or truncated, we corrected the electronic transcripts to reflect the notes taken during each interview verbatim.

Our coding team identified key themes and subthemes inductively through open free coding and analyzed these themes in ATLAS.ti using a content analysis approach.^52^ After initial coding, we checked intercoder agreement by randomly selecting 15 of the 112 transcripts and having a second member of the research team (TL) independently code them using the code book we had developed. We determined 15 transcripts was a sufficient sample to test intercoder agreement because all key themes and subthemes appeared more than once within the sampled transcripts, and many appeared multiple times. After the transcripts of the 15 interviews chosen had been coded by the second coder, the two coders compared how they had applied the codes and discussed the instances in which they had coded the transcripts differently. Based on the consensus the two coders reached about how and why they had applied the codes differently in each instance, the inclusion and exclusion criteria for each code were edited to minimize discrepancy between coders. We then applied the codes with the revised inclusion and exclusion criteria to the remaining 97 interview transcripts.

## Results

### District Health Officer Perceptions and Experiences

We interviewed DHOs (or their proxies) about how health systems and health facilities in their district were managed, including their current situation (2010/2011) and their best recall of two periods in the past (2005/2006, 2007/2008) with regard to the seven health system components described in the Methods section. The goal was to assess whether, during this period of PEPFAR engagement in Uganda, there was a trend towards districts more completely performing a number of key activities to strengthen each health system building block (listed in the column titled “Non-HIV Service Delivery Outcome” of Table 4), and to determine if DHOs perceived PEPFAR had improved, harmed, or had no effect on the health system.

**Table 4:** Uganda DHO Characterization of Health System Components in Their Districts, 2005-2011

**Non-HIV Service Delivery Outcome**	**2005/2006**	**2007/2008**	**2010/2011**
**Planning Capacity**			
Did this district have a strategic plan that covers the year 2011?	98% Yes (but only 43% of these plans were observed) 80% of these included NGOs and 61% included CBOs.		
**M&E**			
Did you use national guidelines on support supervision?	91%	88%	90%
Were private clinics monitored?	69%	65%	68%
Were private labs monitored?	57%	51%	55%
Were private drug shops monitored?	76%	81%	76%
**Resource Mobilization**			
Did this district apply for funds or send out proposal applications beyond the ministry of health (to a non-government funding opportunity)?	31%	44%	49%
**Managing Medical Supplies and Equipment**			
Did you have a district assistant inspector for drugs?	75%	72%	60%
If not, did you have someone formally assigned to conduct drug inspections in the district?	59%	65%	66%
Did you have reports on state of equipment and inventory needs in the district?	65%	76%	76%
**Managing HRs for Health**			
Did the district have a functional service commission?	90%	91%	62%
Did the district have a HRs for health development plan?	63%	66%	84%
Was there a computerized system for tracking the number and movement of health workers (eg, HMIS)?	16%	19%	40%
Was there a “hard to reach” allowance to attract health workers?	7%	21%	23%
Was there a “pay for performance” scheme?	5%	4%	5%
Was there a career development opportunity, such as study leave?	51%	54%	57%
Were there short-term trainings available (eg, workshops)?	58%	61%	67%
Were there top-up allowances or bonuses?	21%	30%	38%
How many technical staff were working in the district office, as a proportion of population? (per 1000 population)	0.020	0.022	0.027
How many support staff were working in the district office, as a proportion of population? (per 1000 population)	0.011	0.012	0.013
Was there a time the DHO position was vacant or filled by an acting appointee between 2005-2011?	No vacancies = 22%, 74% vacant with an acting	
How many DHOs has the district had since 2005?	65% had only 1 (no turnover), 22% had 2, 13% had 3 or more	
**Engaging and Coordinating Stakeholders**			
Did you have a desk officer for coordinating public/private partnerships for health in the district?	22%	24%	42%
Did you conduct any activity to determine the level of community satisfaction with health services in the district?	42%	47%	59%
**Managing Information Systems and Outbreak Investigations**			
Did the district have a staff person with a degree in statistics or biostatistics in charge of HMIS?	9%	31%	45%
If not, did you have someone responsible for analyzing routine data in the district?	97%	98%	98%
How was routine data stored in the district?			
Electronically	2%	6%	7%
On paper	45%	29%	12%
Both	53%	65%	80%
How was routine data transmitted from the district to Ministry HQ?			
Electronically	6%	27%	56%
On paper	75%	49%	10%
Both	19%	23%	33%
Did your district participate in an outbreak investigation?	20%	46%	42%

Abbreviations: DHO, District Health Officer; HRs, human resources; NGOs, non-governmental organizations; HMIS, health management information system; CBOs, community-based organizations; HQ, headquarters. Percentages displayed reflect the portion of respondents answering “Yes” unless otherwise noted. Table provided for descriptive purposes. Full quantitative analyses and discussion of statistical tests for trends 2005-2011 provided in Luboga et al.^50^

Ugandan DHOs generally reported the PEPFAR investment from 2005-2011 was helpful for the country’s overall health system. When asked to categorize PEPFAR’s overall effects on the health system as beneficial, detrimental, or having had no effect, 79% of respondents asserted the effects were beneficial, 8% claimed the effects were detrimental, and 9% responded there were no net effects. An additional 4% either did not know or reported mixed effects (Table 3).

**Table 3:** Uganda DHO Views of the Effects of PEPFAR on Non-HIV Health System Components

	**Planning Capacity (%)**	**M&E Capacity (%)**	**Mobilize Resources (%)**	**Manage Medical Supplies (%)**	**HR Capacity (%)**	**Coordinate Stakeholders (%)**	**Manage Information (%)**	**Total (%)**
Improved	93 (83.0)	91 (81.3)	51 (45.5)	100 (89.3)	88 (78.6)	103 (92.0)	94 (83.9)	620 (79.1)
No effect	8 (7.1)	7 (6.3)	33 (29.5)	7 (6.3)	4 (3.6)	5 (4.5)	7 (6.3)	71 (9.1)
Decreased	5 (4.5)	12 (10.7)	25 (22.3)	2 (1.8)	12 (10.7)	2 (1.8)	3 (2.7)	61 (7.8)
Mixed	3 (2.7)	1 (0.9)	1 (0.9)	2 (1.8)	7 (6.3)	1 (0.9)	1 (0.9)	16 (2.0)
Do not know	3 (2.7)	1 (0.9)	2 (1.8)	1 (0.9)	1 (0.9)	1 (0.9)	7 (6.3)	16 (2.0)

Abbreviations: DHO, District Health Officer; PEPFAR, President’s Emergency Plan for AIDS Relief; HR, human resource; M&E, monitoring and evaluation. Data source: Interviews with Ugandan DHOs (or their proxies) in 112 districts between October and December, 2011. Answer choices offered to the respondents were the five in the table: improved, no effect, decreased, mixed, or do not know.

Examining DHO perceptions of PEPFAR’s effects on each health system component individually, respondents in large numbers (78%-92%) reported PEPFAR’s effects as beneficial for six of the seven health system components studied: *planning capacity, M*&*E capacity, management of medical supplies, human resource (HR) capacity, stakeholder coordination,* and* information management*.

#### Planning Capacity

Almost all (98%) DHOs reported they had a strategic plan in 2011, and 80% of these plans included the activities of NGOs working within the district (Table 4). Overall, 85% of DHOs said PEPFAR improved their ability to plan (Table 3).

#### Monitoring and Evaluation

Only about half of DHOs said private labs were monitored, but 2/3 said private clinics were supervised and 3/4 said private drug shops were monitored. These figures did not change substantially over the period of PEPFAR investment, though 81% of DHOs said HIV initiatives improved their ability to monitor and evaluate non-HIV services in their districts.

#### Resource Mobilization

PEPFAR intended to increase the capacity, interest, and success of host country health leadership in seeking additional third-party funding to improve ownership and sustainability.^53^ Half (49%) of DHOs said they had applied for funds to support their operations beyond the MoH, from either an NGO or other donor, up from about a third at the start of PEPFAR programming. However, only about half (46%) said PEPFAR improved their ability to mobilize resources in their districts, an additional one in five (22%) said it decreased their ability to do so, and a third (30%) said it had no effect. DHOs predominantly attributed this mixed effect on *resource mobilization* to the unavailability of grants for non-HIV related health programs as well as the perception that the windfall of HIV funding had sufficiently provided for the entire health sector (Table 5).

**Table 5:** Uganda DHO Views on Positive and Negative Effects of PEPFAR on Health System Components

**Improved Non-HIV Health Services**	**Health System Building Blocks**	**Weakened Non-HIV Health Services**
• Financial support for planning meetings (33) • More HIV programs incorporated into the planning process (33) • Additional trainings and mentorship for planning (32) • Technical consultation for planning provided to DHO (23)	**Planning Capacity**	• HIV programs do not share work plans with DHO (34) • HIV program work plans too narrowly focus on HIV (32) • Planned projects will not be sustained when HIV programs close (16) • DHO and funder budget cycles differ (6)
• M&E for non-HIV integrated into HIV monitoring (59) • M&E training conducted (35) • HIV organizations carry out or directly fund non-HIV M&E (24) • Additional transport available for non-HIV monitoring visits (9)	**Monitoring and Evaluating non-HIV Programs**	• Too much funding for, and focus on, HIV M&E (37) • DHO staff overwhelmed by additional M&E responsibilities (14) • DHO does not receive M&E reports from HIV organizations (10) • Scaled-up M&E will not be sustained when HIV programs close (4)
• Technical help provided to identify and pursue non-HIV grants (25) • Resources provided by HIV programs also used for non-HIV programming (11) • Additional funding for HIV work allows DHO to dedicate own funds to non-HIV work (10) • Help identifying program gaps and effectively allocating funds (9)	**Resource Mobilization for non-HIV**	• Available grants focused too narrowly on HIV (32) • Health sector misperceived as having sufficient funding (21) • DHO sits back and waits for funders to come (12) • Development partners outcompete DHO for available grants (7)
• Training provided in use and maintenance of medical equipment (80) • Direct provision of medical equipment and supplies (74) • Transport for medicines and diagnostic specimens (20) • Renovation of labs, storage areas, and waiting shades (13)	**Management of Medical Supplies and Equipment**	• Dependence on donors for drugs and supplies (11) • Maintenance of scaled-up diagnostic and treatment services will not be possible when HIV programs close (10) • Push systems deliver drugs and supplies that are not needed (9) • Demand for services has risen with increased medical supply availability (2)
• Training and mentoring improves capacity and builds morale (75) • HIV programs hire and pay additional staff to work at DHO (50) • Opportunity for additional allowances motivates work (46) • Improved work space and equipment boosts morale (15)	**HRs for Health**	• Increased workload and stress (46) • Staff loss to NGO programs (45) • Absenteeism to attend trainings (29) • Damaged morale of staff not working on HIV (19)
• Funding and facilitation of stakeholder meetings provided (86) • Trained and equipped village health teams and community groups (32) • Funding and production of health-themed radio programs (19) • HIV programs assist the formation of coordinating committees and forums (15)	**Coordination of Stakeholders**	• Those attending coordinating meetings now expect to be paid (9) • DHO will not be able to sustain coordination effort when HIV programs close (9) • DHO has insufficient funds to coordinate all stakeholders in the district (6) • HIV programs do not participate in coordination meetings (5)
• Training and capacity building in data capture and analysis provided (83) • Provision of computers, internet, and data storage (80) • Transport and allowances provided for data collection (19) • HIV programs provided forms and registers for data collection (17)	**Management of Information**	• Increased data collection workload (22) • Individual reports needed for each HIV program (21) • DHO does not receive data collected by HIV programs (20) • HIV data collection overly emphasized (13)

Abbreviations: DHO, District Health Officer; HRs, human resources; NGO, non-governmental organization; PEPFAR, President’s Emergency Plan for AIDS Relief; M&E, monitoring and evaluation. Data source: Interviews with Ugandan DHOs (or their proxies) in 112 districts between October and December, 2011. Number of respondents citing each effect in parentheses.

In comparison, very few DHOs (3%) said PEPFAR had harmed *management of medical supplies, ability to coordinate stakeholders*, and *information management capacity*. Slightly more DHOs (5%) reported a negative effect on *planning capacity*, and 11% said PEPFAR had been detrimental both to *M*&*E capacity* and *HR capacity*.

#### Management of Medical Supplies

Though nearly 90% of DHOs said HIV programs had improved their district’s ability to manage medical supplies, the portion of districts reporting the completion of key activities changed modestly 2005-2011. Three-quarters (76%) of DHOs said they had received reports detailing the state of medical equipment and inventory needs in 2010/2011, up from 65% in 2005/2006. During this same period, the percentage of districts with a designated inspector for drugs dropped from 75% to 60%.

#### Human Resources

Most DHOs (90%) said they used the national guidelines for supportive supervision, and their district had been doing so since before PEPFAR started. Similarly, 84% said they had a plan for developing HR capacity at the time of the interview (2011).

#### Stakeholder Coordination

About 42% of DHOs said their districts now had a desk officer for coordinating public/private partnerships, whereas only 22% had such a position at the start of PEPFAR. Also, 59% said they had conducted activities to determine levels of community satisfaction in 2010/2011, up from 42% in 2005/2006.

#### Information Management

The biggest changes over the PEPFAR investment period were in the area of information systems. Whereas at the start of PEPFAR, only 9% had a staff person with a degree in statistics in charge of the HMIS, by 2011, almost half (45%) said they did. Data were routinely transmitted to the Ministry’s Resource Center electronically by almost all (89%) districts, whereas only half (55%) did so in 2007/2008. Fully 90% of DHOs said HIV programs improved their management of information systems.

### Responses to Open-Ended Questions

After asking DHOs to categorize the overall effects of PEPFAR on health system components as positive, negative, or neutral, we prompted them to offer comments in response to open-ended questions about specific positive and negative influences they had experienced. Staff capacity building, integration of HIV and non-HIV services, and improved access to medical supplies and funding emerged as salient positive themes. Key negative themes included vertical HIV programming linked to HIV-specific targets and objectives, set before arriving at the district level, leaving DHOs with little flexibility to address non-HIV health priorities, increased workload, and loss of health service staff to NGOs providing HIV services. Post hoc analyses revealed no discernible differences in the perception of PEPFAR between respondents with different professional titles or duration of tenure in the district (Table 2).

### Positive Effects of PEPFAR Funding: Capacity Building

When we asked open-ended questions about the specific means by which PEPFAR had a positive effect on the non-HIV health system, DHOs most often named training, mentoring, and capacity building as the key themes which cut across all components studied. The greatest number of DHOs said the most important positive effect of PEPFAR was on *HR capacity*, specifically the skills health workers gained. Two in three respondents said trainings had increased the ability of health workers to manage both HIV and non-HIV healthcare at levels ranging from Village Health Teams to skilled service providers. In the words of one respondent, HIV programs and district health offices “built capacity of staff in different skills making them competent in-service delivery not only for HIV, but also in non-HIV service delivery.” Beyond the component of *HR capacity*, many DHOs said additional training improved the *management of medical supplies* as well, outweighing in importance even the direct provision of medical resources. Moreover, many DHOs reported trainings were helpful across all other health system components. Trainings intended to improve M&E of HIV activities also provided skills that could be applied to tuberculosis (TB) control programs. Likewise, skill sets developed to manage pharmacies and forecast stocks of HIV medications also strengthened the supply chain for other essential medicines.

### Positive Effects of PEPFAR Funding: Integration

After training, the second most often named positive effect was the opportunity to integrate HIV and non-HIV program activities. Respondents reported this integration of services as most important to the components of *M*&*E capacity* and* planning capacity*. Integration allowed district health offices to supervise HIV services concurrently with non-HIV services using the same transportation and staff, and using forms and registers which included indicators necessary for the evaluation of both programs:* “They [implementing partners] supported the district by funding the monitoring exercise of HIV programs; in turn, our staff used the opportunity to evaluate other district programs,*” summarized one respondent. DHOs also reported positive effects on *planning capacity,* emphasizing program integration improved the ability of district health offices to plan together with various implementing partners working in the district. One DHO said, “HIV/AIDS organizations have increased multi-sectoral collaboration which has led to a more integrated planning system for the district.” In addition to the particular importance of integration for the components of *M*&*E capacity* and* planning capacity*, respondents also said the integration of HIV and non-HIV was important to all other components of the health system because district health offices could use medical supplies, transport, and funding for staff allowances procured through HIV programs to support non-HIV services as well.

### Positive Effects of PEPFAR Funding: Supplies and Funding

Thirdly, DHOs said the direct provision of medical supplies, transport, and staff allowances, whether through integration of HIV and non-HIV programs or direct support for non-HIV programs, was a major positive effect of PEPFAR. This additional support for non-HIV activities was particularly important to the components of *information management*, *management of medical supplies*, and *HR capacity*. However, a sizable minority of DHOs also cited positive effects of directly supplied resources on *resource mobilization* and *M*&*E capacity*. Comments about the material support provided to bolster *information management* focused primarily on the provision of computer hardware, software, and internet connectivity at health centers, hospitals, and district health offices. Respondents said these resources helped data analysis and timely report submission. DHOs also reported direct material support fortified *management of medical supplies* via the provision of a wider range of equipment. These supplies included microscopes and other lab equipment, refrigerators for cold chain expansion, reagents for diagnostic tests, and buffer stocks of sundry medications for the treatment of malaria, TB, and other bacterial infections.

### Negative Effects of PEPFAR Funding: Vertical Programming

Many DHOs reported PEPFAR’s vertical nature and narrow focus came at the expense of, or did not sufficiently address, public health priorities other than HIV. Many DHOs critiqued the verticality of HIV programs, while acknowledging beneficial spillover of resources. DHOs said the narrow focus of programming was most acutely damaging to the health system components of *resource mobilization*, *M*&*E capacity*, and *planning capacity*. Respondents said *M*&*E capacity* and *planning capacity* were health system building blocks which both benefited by integration of HIV and non-HIV programming while also suffering from vertical HIV funding streams. This contradiction highlights the sometimes divergent ways DHOs perceived PEPFAR’s effects and suggests *M*&*E capacity* and *planning capacity* may be health system building blocks that experienced the most variable influence. A noteworthy minority of respondents commented on how few grant opportunities were available for non-HIV programming as well as the high number of monitoring reports required for HIV activities. Perhaps, though, the most counterproductive effect of PEPFAR mentioned by DHOs attributable to an overemphasis on HIV centered on how PEPFAR funding and support from NGOs changed the political and behavioral landscape in which district health offices operated. Addressing the ability of district health offices to advocate for more funding from the finance ministry, one respondent stated, the:

"*Wrong perception [was] created that the health department has a lot of money because of many HIV activities, which makes it difficult for central and local government to allocate [additional] resources*."

To complicate the matter further, in some cases district health offices themselves changed their resource seeking activities to become more passive and defer to NGOs:

"*The district is not writing proposals for non-HIV services funding because they are being ‘spoon-fed’ by HIV organizations, ie, they expect funds whether they [district health offices] apply or not*."

The fact that health system leaders were more likely to say PEPFAR had harmed or had no effect on their *resource mobilization* than any other component underscores the importance of both of these challenges (Table 3).

###  Negative Effects of PEPFAR Funding: Increased Workload

DHOs reported the implementation of PEPFAR increased the workload for an already overburdened health workforce. With the scale-up of HIV services, district health offices faced the consequences of their own success, seeing an increase in the number of patients seeking care for HIV and non-HIV alike. Respondents attributed this phenomenon to a popular, albeit vague, understanding that all health centers had scaled-up all health services. At the same time, DHOs said their offices were saddled with the burden of additional M&E, data analysis, and reporting to a variety of implementing partners, which often required unique and frequent reports. Meanwhile, many public sector health providers received a growing number of financially enticing offers to join the staff of NGOs, placing additional pressure on district health offices to fill vacancies:

"*HIV programs limit the district capacity to attract workers [overall] because everyone wants to work for these [HIV] programs. For example, when the district advertises for jobs they get no responses, but HIV programs are flooded with applications when they advertise*."

Thus, in addition to shouldering a heavier burden of work, DHOs reported the additional pressure of counteracting the “glamorization” of HIV programs, as one respondent put it.

### Negative Effects of PEPFAR Funding: Internal Brain-Drain

Health sector leaders said the influx of NGOs recruiting health providers from the public sector with lucrative salaries was one of two ways PEPFAR caused, or at least contributed to, health system fragmentation and instability. Beyond the heavier burden of work and challenges filling vacancies, DHOs also saw the disparity in salaries between NGOs and the public sector as harmful to morale:

"*The monetary benefit [offered to NGO staff] affects other staff who are not working for the HIV organizations. This demotivates them because they see their colleagues benefiting a lot, which also compromises services offered*."

Furthermore, respondents worried programs scaled-up with PEPFAR support would be abandoned by district health offices once funding “dries up.”

### District Health Officer Ideas About Reasons for Strengthening or Weakening of the Health System, 2005-2011

When asked to suggest ways by which PEPFAR may have improved the health system between 2005-2011, DHOs most frequently credited increased staffing levels and improved performance (Table 6). Respondents were particularly positive about improvements in the quantity and quality of health staff, making health centers better places to work and seek medical care. DHOs also frequently named physical infrastructure at health centers and availability of medications and medical supplies as key improvements in health system strength. Interestingly, only 12 of the 112 respondents used terms like “cross-cutting” or “spillover” to indicate the use of funds and resources procured for HIV programs being employed for non-HIV activities. Although the theme *integration of HIV and non-HIV services* surely includes similar concepts, resource spillover or integration seem to be relatively less important potential explanations for improvements in the health system than improved staffing or better access to medical supplies and facilities.

**Table 6:** Uganda DHO Ideas on Potential Causes for Health System Improvements or Declines

**Proposed Reasons for Overall Improvement**	**Proposed Reasons for Overall Worsening**
• Increased staffing levels and performance (70) • Improved infrastructure, better access to health facilities (42) • Better monitoring, management, support supervision (40) • Increased availability of drugs and medical supplies (40) • Increased community awareness, knowledge, demand for services (34) • Staff morale, motivation, commitment, and vigilance (32) • Presence of additional donors/partners not otherwise specified (31) • Better integration of HIV and non-HIV services (24) • Transport provided to service providers (19) • Political support (18) • Use of resources brought by HIV programs for non-HIV, “Cross-cutting,” “Spillover” (12)	• Funding and staff focus overemphasizes HIV (39) • Understaffing^a^ (37) • Insufficient health sector funding^a^ (26) • Increased workload (23) • Data quality issues (22) • Staff loss to NGOs (18) • Low staff morale, motivation (18) • infrastructure at health facilities^a^ (16) • Lack of transport^a^ (15) • Negative community attitudes towards health workers, services^a^ (13) • Absenteeism for trainings, outreaches (13) • Continued stock-outs, lack of supplies^a^ (12) • Poor roads^a^ (12) • Health facilities hard to reach, poorly located^a^ (11)

Abbreviations: DHO, District Health Officer; NGOs, non-governmental organizations; PEPFAR, President’s Emergency Plan for AIDS Relief. Data source: Interviews with Ugandan DHOs (or their proxies) in 112 districts between October and December, 2011. Number of respondents citing each effect in parentheses. ^a^ Indicates underlying conditions rather than effects attributable to PEPFAR programs.

As hypothesized, DHOs suggested an overemphasis on HIV programming was the most likely explanation for any potential negative effects of PEPFAR on the health system. According to one respondent, “the mindset of people handling HIV programs [is] that HIV is presumed to have more funding, and they are less interested in programs other than HIV.” Most respondents, however, pointed to underlying and environmental conditions including run-down health facilities and poor transportation rather than the effect of PEPFAR programs themselves. The largest number of DHOs named understaffing as the most important factor for low performance. They also frequently cited insufficient health sector funding, poor health infrastructure, lack of transport, and negative community attitudes towards the health system. For example, one health sector leader offered the potential explanation that Uganda has:

"*Poor infrastructure for non-HIV services -- like you find health center IIIs [facilties designed to serve catchment area populations up to 20 000 people at the sub-county level] could have been given a mandate to handle deliveries, but due to poor infrastructure they cannot. So, people can’t even access it [routine care for births], and also health personnel don’t want to reside there*."

Though increased workload caused by the scale-up of HIV services and the loss of some healthcare providers to HIV programs surely exacerbated these pre-existing challenges, many respondents stressed underlying infrastructural and health workforce challenges as potential explanations for a hypothetical worsening of the health system.

## Discussion

Our goal was to understand how Ugandan DHOs viewed PEPFAR funding in relation to the overall health system with an emphasis on non-HIV health. Complementary analyses of quantitative indicators gathered from district health office records and reported elsewhere^50^ found no meaningful health system improvement or deterioration. However, our qualitative analysis of DHOs’ perceptions about PEPFAR’s influences found DHOs said PEPFAR generally strengthened the health system by improving medical training, integrating HIV and non-HIV activities, and directly providing additional resources. DHOs’ perceptions were not unanimously positive, and many said PEPFAR had exacerbated the loss of staff to NGOs, overemphasized HIV care, and increased workload. One possible explanation for the discrepancy between the largely positive findings of the qualitative assessment and the quantitative analyses showing no major improvement to non-HIV services utilization is that, working within a health system with limited resources, Ugandan DHOs were prone to view any substantial investment positively regardless of its objective downstream effect on service utilization. Another possibility is that DHOs’ statements reflect improvements to other measures, such as of process or quality of services that were not measured in the quantitative paper but have been demonstrated elsewhere.^54^ While quantitative outcome measures like numbers of pediatric outpatient care visits, TB tests, and in-facility deliveries do not reveal meaningful improvements as a result of PEPFAR,^50^ DHOs may nonetheless be largely satisfied with PEPFAR investments for helping the Ugandan health system to maintain non-HIV healthcare service delivery rates while quadrupling the number of patients on HIV treatment.

The positive and negative reports of DHOs regarding PEPFAR are largely consistent with previous studies examining the effects of HIV initiatives on health system strength. A substantial body of literature demonstrates HIV care has improved as a result of investments in training, health infrastructure, and access to treatment.^1,2,55^ Ugandan DHOs in our study agreed, reporting health system strengthening to the extent that training, integration, and direct provision of medical supplies benefited both HIV and non-HIV programs. Answers suggest that in some circumstances, Ugandan DHOs were able to incorporate non-HIV activity into HIV-focused health system work. This approach took many forms, including sharing transportation for HIV care site visits, leveraging HIV planning sessions to also plan for other healthcare service delivery, and tasking additional staff paid by HIV programs to complete both HIV and non-HIV related work, while still fulfilling the objectives and scope of activities PEPFAR agreed to fund.

Reports have also critiqued the narrowing of national health policies to focus on HIV programs,^5,10,12^ duplicative evaluation requirements,^56^ and doctors moving out of the public sector to work for HIV programs.^4,6,14,57,58^ Ugandan DHOs reported they experienced these challenges and confirmed these factors undermined the strength of the health system.

Uganda’s DHOs reported broad satisfaction with PEPFAR, despite criticism of individual aspects and some negative consequences. The extent to which the intensity of PEPFAR investment in Uganda, as an influence distinct from its implementation strategy, is to credit for the 79% (Table 3) of responses citing an improvement in the health system remains an open question. In each year during the study period, PEPFAR expenditures surpassed Ugandan government funding of the health sector (Table 1). Improvements in health system HR capacity, integration, and access to medical supplies DHOs highlighted are certainly consistent with high intensity investment. Though, such high levels of PEPFAR funding may, or may not, be a necessary condition to receive largely positive reviews from DHOs.

PEPFAR offered a major new source of funding in a weak health system starved for resources. It is hardly surprising, therefore, DHOs would be generally happy to receive those resources as they struggled to organize services for rapidly growing populations with a high burden of disease. At the same time, DHOs were not asked how they would choose to direct the influx of PEPFAR resources for the greatest health benefits, so it is unsurprising they might have some criticisms about decisions regarding PEPFAR implementation made by foreigners and at the national level.

During our interviews we observed DHOs were rather pessimistic about the long-standing weaknesses in health system infrastructure in Uganda including an overburdened health workforce, health facilities in disrepair, and insufficient medical equipment. Uganda’s population growth during the study period is unlikely a confounding factor. No DHO cited overall population growth as a key cause of increased stress on health workforce or facilities, and our quantitative analyses (described in Luboga et al)^50^ found most health service volumes grew only slowly or even declined between 2005-2011. DHOs attributed responsibility for these shortcomings to national policy and did not tend to hold PEPFAR accountable. Instead, they chose to highlight the positive gains PEPFAR offered (eg, increased staffing, facility improvements), even if the quantitative evaluation revealed only marginal strengthening of the health system.^50^

DHOs’ comments also contain a number of insights with PEPFAR policy implications. Though space does not permit listing of all implications, the three that follow are the authors’ synthesis of the largest portion of respondents. They are listed with recognition there may be ongoing efforts to address these issues in Uganda or elsewhere:

- Ugandan DHOs recognize many of the services currently funded by PEPFAR will not be financially sustainable for the Ugandan MoH if PEPFAR funding ended. PEPFAR funding depends on both annual US budget appropriations and periodic five-year congressional reauthorizations. Thus, PEPFAR and other key stakeholders should consider engaging with the Government of Uganda to plan an increasingly sustainable and locally-led HIV-response.
- PEPFAR should consider continuing, and further emphasizing, its coordination with the Ugandan MoH during the annual Country Operational Plan (COP) budget planning exercise to ensure HIV activities are consistent with national priorities and funding pipelines complement each other.
- PEPFAR should consider encouraging the implementing partners through which it works to adopt policies which limit the loss of public sector healthcare workers to private HIV-focused NGOs.^58^

Funding for our research was provided through a PEPFAR Public Health Evaluation award through CDC, a US government agency with a major role in implementing PEPFAR in Uganda. UW co-authors received PEPFAR funding for multiple projects, including this one, and I-TECH, affiliated with UW, was a major implementing partner and recipient of PEPFAR funding in Uganda and elsewhere during the study period. Other limitations include the brief amount of time we spent with each DHO, which limited the depth of responses we received. The interview portions of our visits with DHOs were necessarily succinct. Indeed, our ability to have conversations with health officers in all 112 Ugandan districts, a unique strength of our research, is attributable to the efficiency of each visit. Our choice to rely on handwritten interview transcripts, rather than audio recordings, is also an inherent limitation of this approach. While we can generalize our findings to all of Uganda, observations are still limited to Uganda’s specific political, professional, and economic contexts and do not necessarily apply to the experiences of public sector health leaders with HIV initiatives in other countries. In many cases, respondents had not been appointed to their job or posted in their current district for the full duration of the 2005-2011 time frame we investigated. During the study period some DHOs were appointed or promoted to pre-existing districts, and others rose to DHO positions as Uganda decentralized its public health system and split 56 districts into 112. We did not ask respondents to comment specifically on periods prior to their current placement and this analysis cannot assess how PEPFAR’s influence may have varied by district over the years. Additionally, the retrospective study design may have induced recall bias. DHOs may have been reticent to appear unappreciative of the significant financial contributions of PEPFAR by expressing criticisms and in some cases may have benefited personally from PEPFAR themselves, for example, through receiving per diem allowances to attend PEPFAR-supported trainings. Moreover, DHOs’ knowledge that our study was funded by PEPFAR may have limited expression of criticism. Therefore, our results may have under-reported negative perceptions of PEPFAR and over-reported positive perceptions. To minimize the likelihood this would happen, we informed respondents their opinions and statements would not be attributed to them personally and their names would not be used in data analyses or reports. We also conducted all interviews in private settings and trained the data collection teams to establish collegial rapport with respondents before beginning the interviews. Lastly, though PEPFAR comprised the large majority of Uganda’s budget for HIV/AIDS in during the study period, it is difficult to parse the discrete contributions of the various GHIs supporting HIV/AIDS programming in Uganda between 2005-2011. DHOs frequently mentioned PEPFAR by name in their responses, but it remains difficult to attribute specific results to the effects of a single funding stream.

## Conclusion

Ugandan DHOs reported PEPFAR strengthened their health systems between 2005-2011. However, DHOs were not unanimously or uniformly positive. While the overall satisfaction rate with PEPFAR approached 80% positive ratings among DHOs, fewer than half reported an improvement in their ability to mobilize resources to strengthen the health system beyond HIV services. These challenges are neither new nor unique to the Ugandan context. As PEPFAR proceeds into its third phase, focusing on sustaining control of the HIV epidemic while gradually transferring leadership to ministries of health, increased emphasis has been placed on improved government engagement and health system strengthening.^59,60^ Still, the goals of PEPFAR-supported health system strengthening, and efforts to develop HRs for health, remain HIV-focused.

DHOs also offered constructive criticisms of PEPFAR’s effects on other health system components. They tended to credit improvements in health system strength to PEPFAR’s influence, while attributing declines to pre-existing shortcomings in health system infrastructure and workforce. This tendency is consistent with DHOs’ positive perception of PEPFAR’s effects, despite modest evidence for increased health service utilization from separate quantitative analyses.^8,14,16,50,61^ As HIV infection becomes a chronic disease requiring strong health systems to manage sustained patient care over time, Uganda’s weak health systems will require broad infrastructure development inconsistent with narrow vertical health programming. DHOs expressed significant concerns about what will happen to health system advances funded by HIV programs once PEPFAR ends. Ultimately, any improvements to either HIV or non-HIV health service delivery systems will depend almost entirely on the sustainability of activities undertaken with funding from PEPFAR and other donors. Nonetheless, health system leaders in Uganda at the district level were appreciative of resources aimed at HIV they could often leverage for broader purposes.

## Acknowledgements

This study was conducted with data provided by the MoH of the Republic of Uganda, Kampala, Uganda with support from the CDC-supported Prevention Research Center, Schools of Medicine and Public Health, University of Washington, Seattle, WA, USA. We appreciate the additional contributions to the cost of data collection by the College of Health Sciences, Makerere University, Kampala, Uganda and the Schools of Medicine and Public Health, University of Washington, Seattle, WA, USA. We appreciate the two-dozen Ugandan team members who travelled throughout the nation to collect data (often in hazardous conditions), and the many Ugandan clinic and district staff persons who carefully competed their routine data reports for us to collect. Thanks to Aida Namubiru and Evelyn Bakengesa for their administrative assistance.

Research funding was provided by PEPFAR through the CDC under the terms of the Public Health Evaluation project entitled “Assessment of the Impact of PEPFAR/Global Disease Initiatives on non-HIV Health Services and Systems in Uganda” [grant number CE.08.0221]. While the CDC cleared this manuscript for publication, the findings and conclusions in this report are those of the authors and do not necessarily represent the official position of CDC.

## Ethical issues

Institutional review board approval was obtained from the Uganda National Council for Science and Technology, the Makerere University School of Medicine, Kampala, Uganda and the University of Washington, Seattle, WA, USA. The study protocol went through scientific and technical review and received approval at the division and center level at CDC. All parties signed a data user agreement stipulating that the Uganda MoH owns the data.

## Competing interests

Our study of the health systems effects of PEPFAR was supported with PEPFAR funding. The lead author currently works as a contract employee with the PEPFAR-Mozambique Strategic Information team.

## Authors’ contributions

NL developed the analytical framework and completed analysis of qualitative data with contributions from AH, TL, and JP. NL and AH drafted the manuscript with contributions from SL, BS, TL, FM, NK, FL, AN, EM, SB, and JP. AH, SB, and BS developed the research protocol with contributions from SL, TL, NK, AN, and EM. SL, FM, NK, FL, and EM oversaw data collection and quality and contributed to analysis of quantitative data.

## Authors’ affiliations

^1^Department of Global Health, University of Washington, Seattle, WA, USA. ^2^Department of Health Services, University of Washington, Seattle, WA, USA. ^3^Faculty of Health Sciences, Makerere University, Kampala, Uganda. ^4^Division of Global HIV and Tuberculosis, Atlanta, GA, USA. ^5^Resource Center for the Uganda Ministry of Health, Uganda Ministry of Health, Nakasero, Uganda.

## Key messages

**Implications for policy makers**

- District Health Officers (DHOs) and other public sector health managers have a unique perspective on the massive growth in the number of individuals on HIV care and treatment since 2003. As Uganda and other countries with high HIV prevalence align with the latest World Health Organization (WHO) recommendations and adopt universal HIV test and start treatment strategies, their input will be invaluable to the development of effective and sustainable policies.
- HIV is quickly becoming a chronic infection requiring increased health system capacity to manage an exponential rise in the number of individuals on HIV treatment. It is important to leverage HIV support for broader health system strengthening.
- Policy-makers should consider continuing to monitor the extent to which funding specifically targeted for HIV programs may make it more difficult for public sector health managers to mobilize resources for non-HIV programs.

**Implications for public**

The global community has set a goal to eliminate AIDS by 2030, but doing so will require massively expanding HIV testing and treatment programs. President’s Emergency Plan for AIDS Relief (PEPFAR) and other global health initiatives (GHIs) directly targeting HIV have proven it is possible to scale-up HIV treatment services quickly and effectively. Yet, it remains unclear how growing HIV programs have strengthened and stressed broader health systems. The experience of Ugandan District Health Officers (DHOs) between 2005-2011 illustrates many of the opportunities and challenges public sector health managers will face as HIV programs continue to enroll more people on treatment. It is important the public be engaged in this process of expansion by supporting those on HIV treatment, demanding donors continue to fund treatment for those who cannot afford it, and advocating HIV funding be employed for broader health service delivery. As echoed by the comments of Ugandan DHOs, it will take broad-scale engagement of both HIV-negative and -positive Ugandans to ensure the substantial funding entering Uganda vertically targeted at HIV programs supports sustainable health system improvement.
